# The causal relationship between physical activity, sedentary time and idiopathic pulmonary fibrosis risk: a Mendelian randomization study

**DOI:** 10.1186/s12931-023-02610-3

**Published:** 2023-11-20

**Authors:** Wanyang Lei, Mei Yang, Ziyu Yuan, Runlin Feng, Xiao Kuang, Zhiqiang Liu, Zongqi Deng, Xianglin Hu, Wenlin Tai

**Affiliations:** 1 Department of Clinical Laboratory, Yunnan Molecular Diagnostic Center, The Second Affiliated Hospital of Kunming Medical University, Kunming, Yunnan China; 2 Department of Respiratory and Critical Care, Yunnan Second People’s Hospital, Kunming, Yunnan Province China; 3 Department of Clinical Laboratory Medicine, The Third Affiliated Hospital of Kunming Medical University, Yunnan Cancer Hospital, Yunnan Cancer Center, Kunming, 650118 China; 4 Department of Thoracic Surgery I, The Third Affiliated Hospital of Kunming Medical University (Yunnan Cancer Hospital, Yunnan Cancer Center), Kunming, China

**Keywords:** Idiopathic pulmonary fibrosis (IPF), Physical activity (PA), Sedentary time, Mendelian randomization (MR), Causal effect

## Abstract

**Background:**

Several observational studies have found that physical inactivity and sedentary time are associated with idiopathic pulmonary fibrosis (IPF) risk. However, the causality between them still requires further investigation. Therefore, our study aimed to investigate the causal effect of physical activity (PA) and sedentary time on the risk of IPF via two-sample Mendelian randomization (MR) analysis.

**Methods:**

Multiple genome-wide association study (GWAS) data involving individuals of European ancestry were analyzed. The datasets encompassed published UK Biobank data (91,105–377,234 participants) and IPF data (2018 cases and 373,064 controls) from FinnGen Biobank. The inverse variance weighting (IVW) method was the primary approach for our analysis. Sensitivity analyses were implemented with Cochran’s Q test, MR-Egger regression, MR-PRESSO global test, and leave-one-out analysis.

**Results:**

Genetically predicted self-reported PA was associated with lower IPF risk [OR = 0.27; 95% CI 0.09–0.82; P = 0.02]. No causal effects of accelerometry-based PA or sedentary time on the risk of IPF were observed.

**Conclusions:**

Our findings supported a protective relationship between self-reported PA and the risk for IPF. The results suggested that enhancing PA may be an effective preventive strategy for IPF.

**Supplementary Information:**

The online version contains supplementary material available at 10.1186/s12931-023-02610-3.

## Introduction

Idiopathic pulmonary fibrosis (IPF) imposes a significant economic burden on society, with its global incidence steadily rising yearly [1]. Patients diagnosed with IPF experience a median survival time of fewer than three years, and their mortality rate surpasses that of many malignancies [2]. The lack of established protective factors makes pursuing IPF prevention exceptionally complex [3]. One promising approach to address this challenge is participation in physical activity (PA) and reducing sedentary time [4]. This realm highlights the connection between PA and overall health, which has garnered widespread acknowledgment from medical experts and researchers. PA offers numerous benefits, including reducing the risk of developing chronic diseases and enhancing the management of existing medical conditions [5].

The interplay between PA and IPF has garnered extensive attention in recent years. Various randomized clinical studies have demonstrated the efficacy of PA in ameliorating symptoms and enhancing outcomes in IPF patients [6]. However, it is essential to note that while randomized clinical trials (RCTs) are methodologically robust and minimize confounding variables, they are often carried out on a relatively limited scale [7]. Moreover, these trials primarily focus on symptom management in individuals already diagnosed with IPF rather than assessing PA as a preventive measure in a broader population [8]. Compared to people adopting healthier lifestyles, recent observational studies have unveiled a link between poor lifestyle habits—such as a sedentary lifestyle and physical inactivity—and an elevated risk of developing IPF. Nevertheless, a gap persists in direct evidence that establishes a causal relationship between PA and IPF events [9–11]. The inherent limitations of traditional research designs render existing observational studies unable to negate the potential for reverse causality and confounding factors entirely. These limitations can introduce association bias and subsequently affect the resulting conclusions [12]. In contrast, RCTs offer the advantage of assessing clinical interventions' theoretical efficacy or effectiveness. They are considered the optimal method for mitigating selection bias and confounding in clinical research, making them the gold standard for establishing causation [13]. However, in the context of investigating the causal relationship between PA and the risk of IPF, conducting RCT studies may not be feasible. Despite being a powerful tool, RCTs might not be realistic or ethical for addressing this research question.

Mendelian randomization (MR) offers an alternative approach for potential causal inference when conducting RCTs is either impractical or unfeasible [14]. MR employs genetic variation to establish causal links between exposures and outcomes. Genetic variants are randomly assigned at conception and often remain independent of environmental risk factors [15]. Furthermore, these genetic variants precede the onset of diseases. As a result, MR analysis can exclude the influence of unmeasured confounders [16, 17].

In this paper, we assess the potential cause-effect between PAs, sedentary time and the risk of IPF via MR analysis. Our investigation relies on large-scale genome-wide association study (GWAS) data. The primary objective of this study is to provide valuable insights into preventive intervention strategies for IPF, informed by the outcomes of our MR analysis.

## Methods

### Study design

**Fig. 1:**
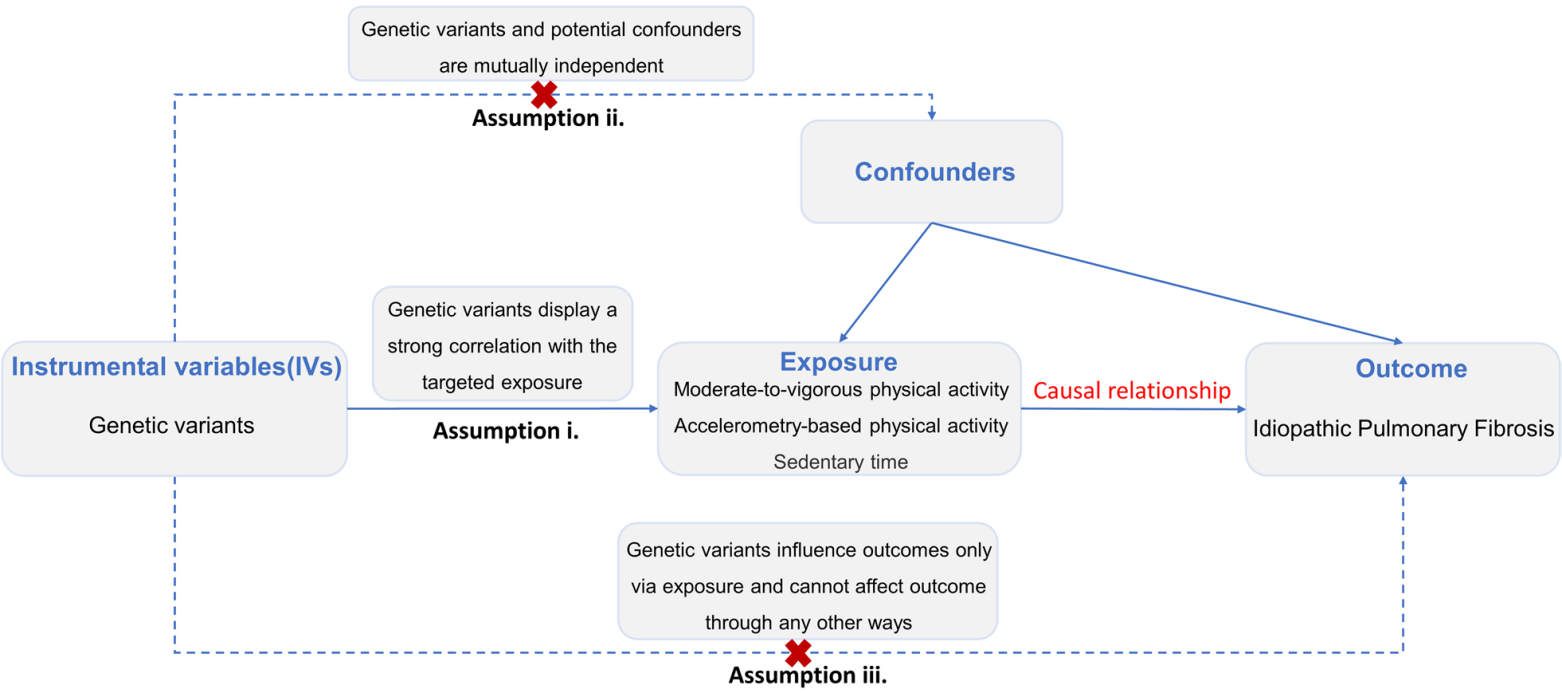
Diagram illustrating the three assumptions of Mendelian randomization

We performed a two-sample MR study to assess the causal effect of PA/sedentary time on IPF using GWAS summary statistics. This instrumental variable (IV) analysis emulates an RCT regarding the random allocation of single nucleotide polymorphisms (SNPs) among offspring, free from confounding factors such as sex and age. In this study, the MR design must adhere to three assumptions [18] (Fig. 1).

### GWAS summary data for physical activity and sedentary time

Summary data for self-reported and accelerometer-based PA phenotypes were obtained from the UK Biobank [19, 20]. The UK Biobank project, a prospective cohort study, encompasses a participant pool of over 500,000 individuals aged between 40 and 69, all within the community setting [21]. For self-reported PA, data were collected from 377,234 UK Biobank participants who provided information about their PA levels through the International PA Questionnaire Short Form. The moderate-to-vigorous PA calculation involved summing the weekly minutes of moderate PA and vigorous PA, and then multiplying this sum by eight [19].

Regarding accelerometry-based PA (“average acceleration”), exercise data were gathered from over 100,000 participants who wore Axivity-AX3 accelerometers on their wrists [22]. After applying essential calibration steps that included removing gravitational effects and sensor-generated noise, and identifying wear versus nonwear intervals, the resulting raw triaxial acceleration data (captured at 100 Hz) were utilized to compute various PA metrics. The exposure variable derived from accelerometer data was labeled “average acceleration” [19]. Additionally, sedentary time data were extracted from the UK Biobank accelerometer data, defined as activity levels equal to or below 1.5 METs (metabolic equivalent of task score) [20].

### Selection of genetic instruments

For the robustness of the MR analysis, it is necessary to consider the effect of vertical and horizontal pleiotropy on MR analysis [23]. Therefore, the choice of IVs for PAs and sedentary time was guided by specific criteria outlined by Martin Bahls and Chen Xiong et al. These criteria encompassed several steps. Initially, SNPs reaching the genome-wide significance threshold (P < 5 × 10^–8^) were included, and SNPs exhibiting significant associations with IPF were excluded. Subsequently, SNPs were clumped (threshold: r^2^ = 0.001, window size: 10,000 kb). Third, SNPs that could exhibit pleiotropic effects were removed [24, 25]. In addition, the F statistic $(F={beta}^{2}/{se}^{2})$ was calculated to tackle potential bias from weak instrumental variables [26]; an F statistic below 10 indicated an invalid instrumental variable. Ambiguous and palindromic SNPs were harmonized to ensure accuracy [27]. Steiger filtering was employed to further refine the selection of SNPs [28]. These comprehensive approaches collectively ensured the integrity and reliability of our MR analysis.

### GWAS summary statistics for idiopathic pulmonary fibrosis

To prevent participant overlap, genomic data of 2018 cases and 373,064 controls were utilized as outcome variables in the GWAS analysis of IPF from the FinnGen Biobank (R9) [29]. IPF cases were identified by searching hospital discharge or mortality records, with a median age at the first occurrence of 71.61 years. IPF was determined by the International Classification of Diseases, ICD-10 codes (ICD-10-J84.1).

### Data availability

The GWAS summary data of PAs and sedentary time can be accessed at the GWAS Catalog (https://www.ebi.ac.uk/gwas/). Additionally, the summary data of IPF can be found at FinnGen Biobank (https://www.finngen.fi/en/access_results). All data were derived from public databases and did not require ethical and moral review.

### Statistical analyses

We employed a two-sample MR method to explore the causal relationship between IPF and PA. The cornerstone of our investigation was inverse variance weighted (IVW) analysis, which served as the primary analytical method. This approach involves a meta-analysis of each SNP's Wald ratio between the exposure and the outcome [30]. If potential heterogeneity across the analyzed SNPs, the analysis uses a random-effects inverse-variance system, and each balance is weighted based on its corresponding standard error [31]. We also employed complementary approaches, including MR-Egger [32], weighted median [33], weighted mode [34], simple mode [35], and MR pleiotropy residual sum and outlier (MR-PRESSO) methods [36]. The MR analysis was conducted via the R environment (version 4.3.1) within the TwoSampleMR (version 0.5.7) and MRPRESSO (version 1.0) packages.

### Sensitivity analyses

A multistep process was undertaken for sensitivity analysis and assessing the second and third assumptions. Cochran’s Q test initially evaluated heterogeneity among the IVs [37]. Subsequently, the MR-Egger regression and MR-PRESSO global test examined the potential horizontal pleiotropy of the IVs [32, 38]. A leave-one-out analysis was also performed to determine if a single SNP with a significant horizontal pleiotropic effect could substantially influence the MR estimates [39].

## Results

In our study, 18 SNPs were employed as IVs for self-reported PA, 6 for accelerometry-based PA, and 5 for sedentary time. The F statistics for all genetic instruments exceeded 29.98, indicating no instrument bias (Additional file 1: Tables S1–S3).

### MR estimates

**Fig. 2:**
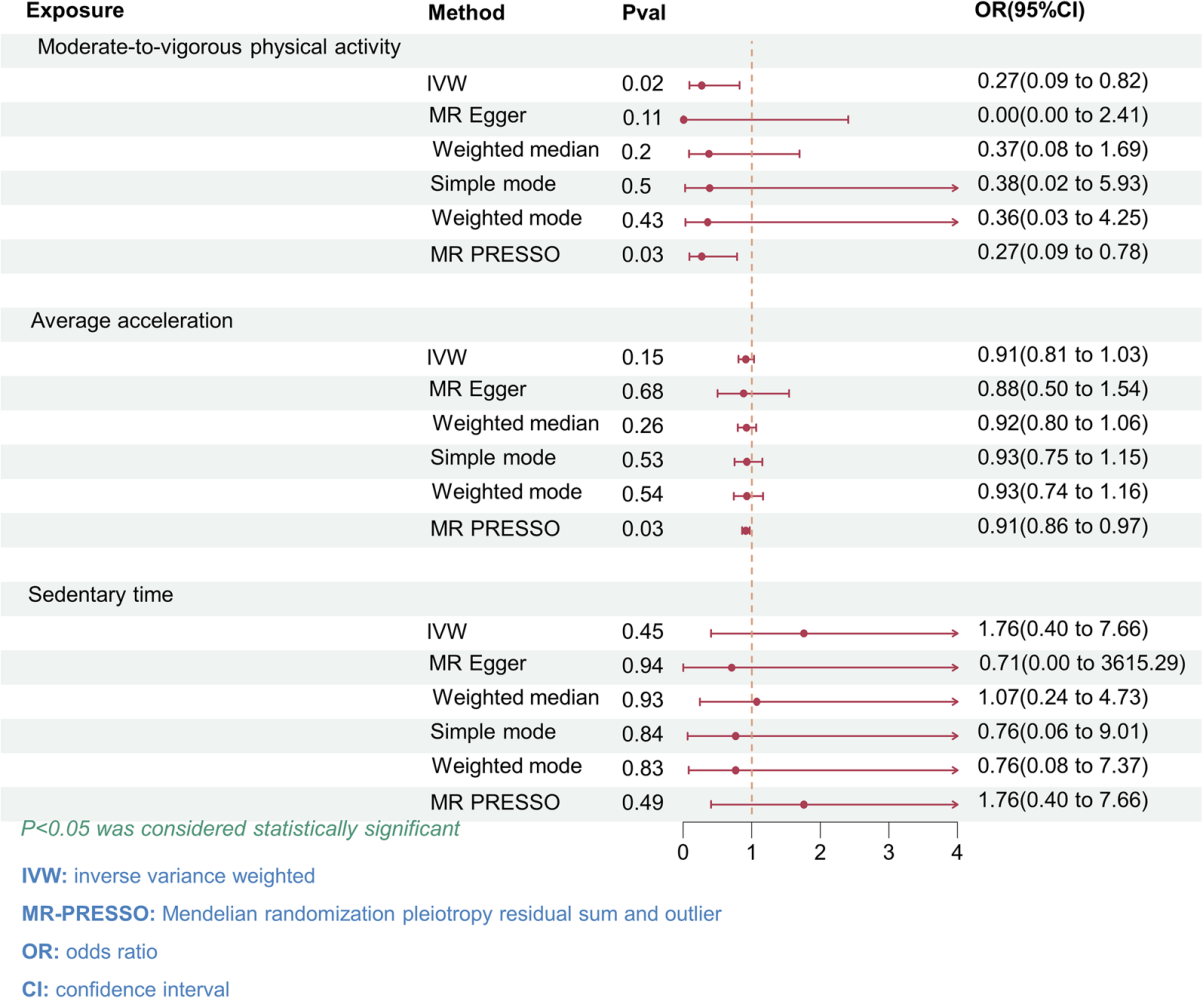
Forest Plot of the Association of Physical Activity and Sedentary Time with IPF

IVW analysis revealed that moderate-to-vigorous PA was associated with a decreased risk of IPF (OR = 0.27, 95% CI 0.09–0.82; P = 0.02) (Fig. 2). The MR-PRESSO regression analysis supported this relationship (OR = 0.27, 95% CI 0.09–0.78; P = 0.03) (Fig. 2). Moreover, the results from other MR methods exhibited a congruent direction, although the observed trends were not statistically significant. For the causal correlation analysis of accelerometry-based PA and IPF, consistent effect direction across all MR methods, the MR-PRESSO regression analysis supported that accelerometry-based PA was associated with a decreased IPF risk (OR = 0.91, 95% CI 0.86–0.97; P = 0.03), but IVW analysis did not indicate such a relationship (OR = 0.91, 95% CI 0.81–1.03; P = 0.15) (Fig. 2). Our analysis did not reveal any causal relationship between genetic predisposition for sedentary time and the risk of IPF.

### Sensitivity analyses

All P values of the Cochran Q tests were > 0.05, indicating no apparent presence of heterogeneity (Table 1). A small number of potential outliers of SNPs can be seen in scatter plots and leave-one-out plots (Additional file 2: Figs. S1–S3). Nevertheless, there was insufficient evidence for a horizontal pleiotropic relationship between PA phenotypes, sedentary time and IPF, as all P values of the further Egger intercept tests MR-PRESSO global tests were > 0.05 (Table 1).

**Table 1:** Pleiotropy and heterogeneity tests of MR

Exposure	Test	Method	Effect size	P
Moderate to vigorous physical activity	Heterogeneity	Cochran's Q test (MR Egger)	1.131	0.89
		Cochran's Q test (IVW)	1.149	0.95
	Pleiotropy	MR-Egger regression (egger_intercept)	0.009	0.9
		MR-PRESSO global test	1.687	0.95
Average acceleration	Heterogeneity	Cochran's Q test (MR Egger)	13.976	0.6
		Cochran's Q test (IVW)	15.685	0.55
	Pleiotropy	MR-Egger regression (egger_intercept)	0.062	0.21
		MR-PRESSO global test	17.806	0.56
Sedentary time	Heterogeneity	Cochran's Q test (MR Egger)	7.335	0.06
		Cochran's Q test (IVW)	7.447	0.11
	Pleiotropy	MR-Egger regression (egger_intercept)	0.028	0.84
		MR-PRESSO global test	11.389	0.18

*MR* Mendelian randomization, *IVW* inverse variance weighted

## Discussion

IPF, a progressive lung disorder characterized by the scarring of lung tissues, remains without a definitive cure, according to our current understanding [1–3]. While completely eradicating the condition may be beyond our current capabilities, the focus can shift toward reducing the probability of its occurrence. Individuals can adopt proactive measures to mitigate the risk and impact of IPF. A proactive approach centered on prevention becomes a critical pathway. Therefore, delving into identifying protective factors associated with IPF becomes imperative, as it can bolster prevention and early intervention strategies for this condition.

The results demonstrated that moderate-to-vigorous PA significantly reduces the risk of IPF. The IVW analysis did not reveal a causal association between accelerometry-based PA and IPF risk. However, a consistent effect direction was observed across all MR methods employed, and significant results were found in the MR-PRESSO regression analysis. These results indicated that accelerometer-based PA tends to reduce the risk of IPF. Consistent with our results, an observational prospective study conducted in Israel highlighted that engaging in regular PA, specifically walking for more than 150 min per week, led to a remarkable 74% reduction in the risk of hospitalization among 34 patients with IPF [9]. Furthermore, another follow-up study involving IPF patients indicated that even engaging in low levels of PA, ranging from 100 to 105 min per week, yielded positive outcomes by reducing mortality rates and enhancing the prognosis of IPF [10]. A large cohort study that investigated the interplay between lifestyle, genetic predisposition, and susceptibility to developing IPF revealed significant findings. It was observed that individuals diagnosed with IPF exhibited a lower likelihood of participating in regular PA [11]. While these studies indicate a correlation between PA and a reduced risk of IPF, they do not definitively elucidate the causal impact of PA on IPF risk. Compared to extensive prospective clinical trials that demand prolonged observation periods, which are relatively impractical, MR studies present a notable reduction in both time and cost and offer a novel perspective on the causal connection between PA and IPF [17].

Interestingly, our study revealed no indication of a causal relationship between sedentary time and the risk of IPF. This finding contradicts previous observational studies that have explored the link between sedentary time and IPF risk. For instance, a prospective study highlights a potential link between sarcopenia and the disease among individuals diagnosed with IPF. Notably, this observation suggests that patients with sarcopenia are more likely to adopt a sedentary lifestyle [40]. Another study revealed that elevated risks were evident when contrasting patients who reported a daily sitting time of less than 5 h with those who spent 5 to less than 10 h and those who sat for 10 h or more each day. Specifically, there was a 2.4-fold and 5.8-fold increase in risk (P trend = 0.036) for hospitalization and a 4.6-fold and 21.2-fold increase in risk (P trend = 0.018) for mortality, respectively [9]. While these studies suggest a correlation between sedentary time and IPF, these observational findings are susceptible to confounding variables and the possibility of reverse causality. In contrast, our study distinguishes itself by providing causal evidence of the role of low sedentary time in diminishing the risk of IPF within the framework of an MR design.

PA offers immediate advantages, such as a lower risk of obesity [41] and diabetes [42]. Furthermore, extensive research has considered obesity [43] and diabetes [44] as risk factors associated with IPF. Obesity may act as an intermediate link between PA and IPF. Obesity is linked to various adverse effects, including reduced lung function [45], chronic lung inflammation [46], and a high incidence of gastroesophageal reflux disease (GERD) [47]. Observational data suggested that as many as 90% of individuals with IPF exhibit abnormal acidic GER [48]. Several subsequent MR studies have confirmed that GERD is crucial in elevating the risk of IPF [49, 50]. Persistent inflammation is deemed significant in developing lung fibrosis, which may contribute to the occurrence of IPF [51]. For example, the expression of tumor necrosis factor-alpha (TNF-α) was upregulated in bleomycin-induced mouse lung tissue, whereas mice with TNF-α receptor knockout exhibited protection from asbestos-induced fibrosis [52, 53]. MR results suggest that elevated circulating C-reactive protein (CRP) levels may be associated with an increased risk of developing IPF [54]. A comprehensive meta-analysis encompassing five studies and 275 participants highlighted the positive impact of exercise interventions on CRP levels in older adults undergoing hospitalization [55]. Another meta-analysis encompassing 14 RCTs among individuals with diabetes corroborated the favorable effects of aerobic exercise on the levels of the inflammatory biomarkers CRP and TNF-α [56]. These studies offer a potential rationale for the causal relationship between PA and a reduced risk of IPF. Regular PA may lower the risk of developing IPF by impacting chronic diseases and the inflammatory state.

The study at present is the first to assess whether PA could decrease the risk of IPF in a two-sample MR framework, which effectively exclude the effect related to unobserved confounding and reverse causality. Moreover, our study underwent rigorous sensitivity analyses to assess the validity of the MR assumptions, thereby minimizing the potential for biased outcomes. If IVs can directly affect the result without via exposure, which indicates the existence of horizontal pleiotropy. The IVW method, as the primary approach, provided enhanced statistical power compared to other MR methods. However, it is also the most demanding method, requiring that all SNPs do not with horizontal pleiotropy, so the results will be easily affected by potential horizontal pleiotropy (if any) [57]. We used the MR-Egger regression and the MR-PRESSO global test to ensure that SNPs had no potential pleiotropy, which guarantees the robustness of our analysis results. The lack of statistical significance and wider confidence intervals in the MR-Egger results can be attributed to its lower statistical power, which is to be anticipated [58]. Our study diligently upheld the prerequisite of consistent beta direction across all MR methods, reinforcing the reliability of our findings. However, certain limitations warrant consideration. First, the generalizability of our findings to other ethnic groups with distinct cultures and lifestyles may be limited due to the European ancestry of the dataset participants. Second, IPF cases of GWAS were identified by mortality/discharge records, which may affect the reliability of the diagnosis. Third, the complete exclusion of pleiotropy remains challenging, given the incomplete understanding of the functional biological roles of the genetic variants. Finally, while our results offer insight into potential causal relationships between PA and IPF risk, further exploration is necessary to investigate the underlying mechanisms of these associations.

## Conclusions

In conclusion, through the MR analysis of large-scale GWAS data, our study has bolstered the evidence indicating a causal link between PA and the risk of IPF. Additional research is needed to elucidate the underlying mechanisms connecting PA with IPF. Considering the imperative of preventing IPF, significant emphasis should be placed on lifestyle management, including promoting regular exercise as a relevant strategy for preventing IPF.

### Supplementary Information

**Additional file 1: Table S1.** Instrument variables of Moderate-to-vigorous physical activity. **Table S2.** Instrument variables of Average acceleration. **Table S3.** Instrument variables of Sedentary time.

**Additional file 2: Figure S1.** Scatter plot (A), Funnel plot (B), forest plot (C) and leave-one-out analysis (D) for moderate-to-vigorous physical activity on idiopathic pulmonary fibrosis. **Figure S2.** Scatter plot (A), Funnel plot (B), forest plot (C) and leave-one-out analysis (D) for average acceleration on idiopathic pulmonary fibrosis. **Figure S3.** Scatter plot (A), Funnel plot (B), forest plot (C) and leave-one-out analysis (D) for sedentary time on idiopathic pulmonary fibrosis.

## Data Availability

All data generated or analysed during this study are included in this published article and its Additional files.

## Abbreviations

IPF: Idiopathic pulmonary fibrosis
PA: Physical activity
RCTs: Randomized clinical trials
MR: Mendelian randomization
GWAS: Genome-wide association studies
IV: Instrumental variable
SNPs: Single nucleotide polymorphisms
IVW: Inverse variance weighted
MR-PRESSO: MR pleiotropy residual sum and outlier
GERD: Gastroesophageal reflux disease
TNF-α: Tumor necrosis factor-alpha
CRP: C-reactive protein
