# Supplementary material for: The causal relationship between physical activity, sedentary time and idiopathic pulmonary fibrosis risk: a Mendelian randomization study
Source: Respir Res. 2023 Nov 20;24:291. doi: 10.1186/s12931-023-02610-3 (PMC10658800; doi:10.1186/s12931-023-02610-3)
Supplement: Supplementary file 2 — Additional file 2: Figure S1. Scatter plot (A), Funnel plot (B), forest plot (C) and leave-one-out analysis (D) for moderate-to-vigorous physical activity on idiopathic pulmonary fibrosis. Figure S2. Scatter plot (A), Funnel plot (B), forest plot (C) and leave-one-out analysis (D) for average acceleration on idiopathic pulmonary fibrosis. Figure S3. Scatter plot (A), Funnel plot (B), forest plot (C) and leave-one-out analysis (D) for sedentary time on idiopathic pulmonary fibrosis. [file 12931_2023_2610_MOESM2_ESM.pdf]

Supplementary Figures

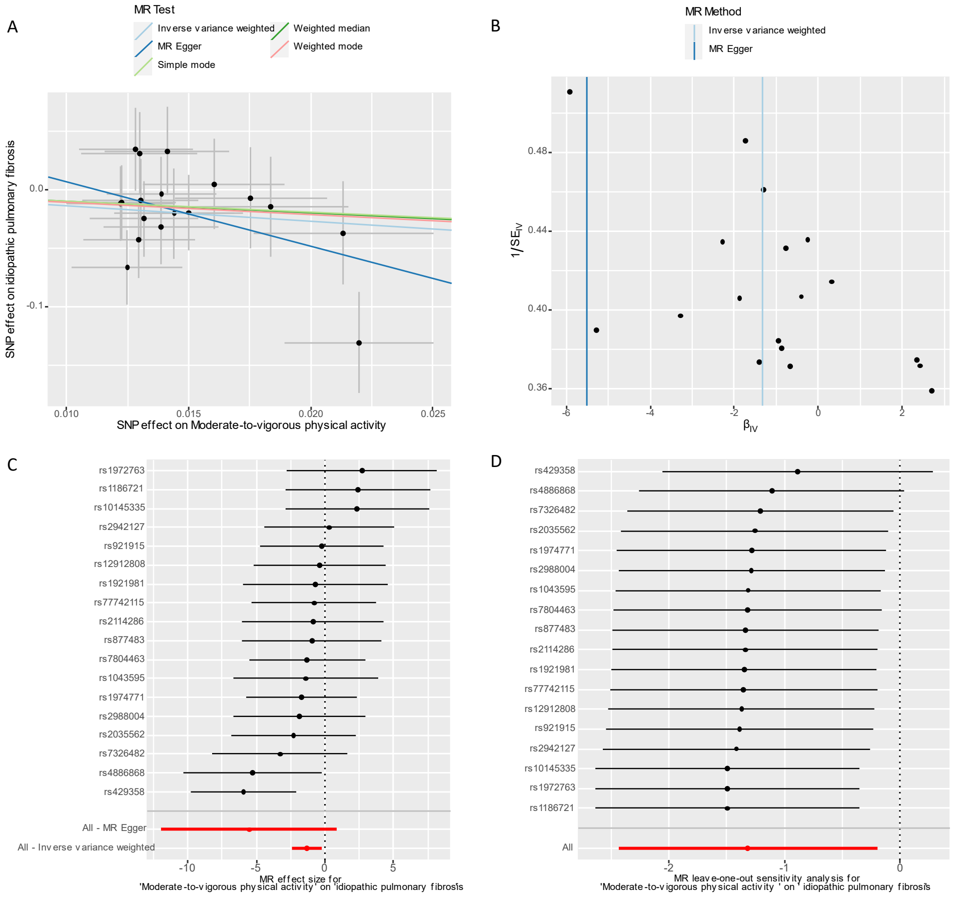

Figure S1. Scatter plot(A), Funnel plot(B), forest plot(C) and leave-one-out analysis(D) for moderate-to-vigorous physical activity on idiopathic pulmonary fibrosis.

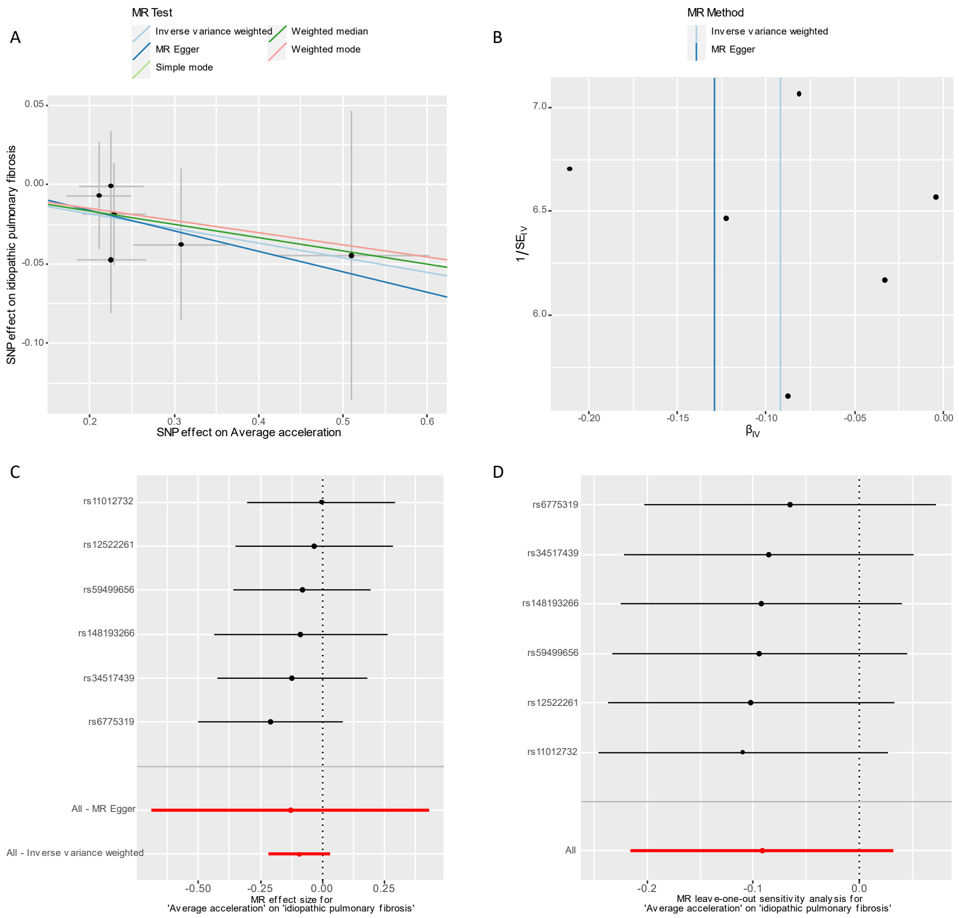

Figure S2. Scatter plot(A), Funnel plot(B), forest plot(C) and leave-one-out analysis(D) for average acceleration on idiopathic pulmonary fibrosis.

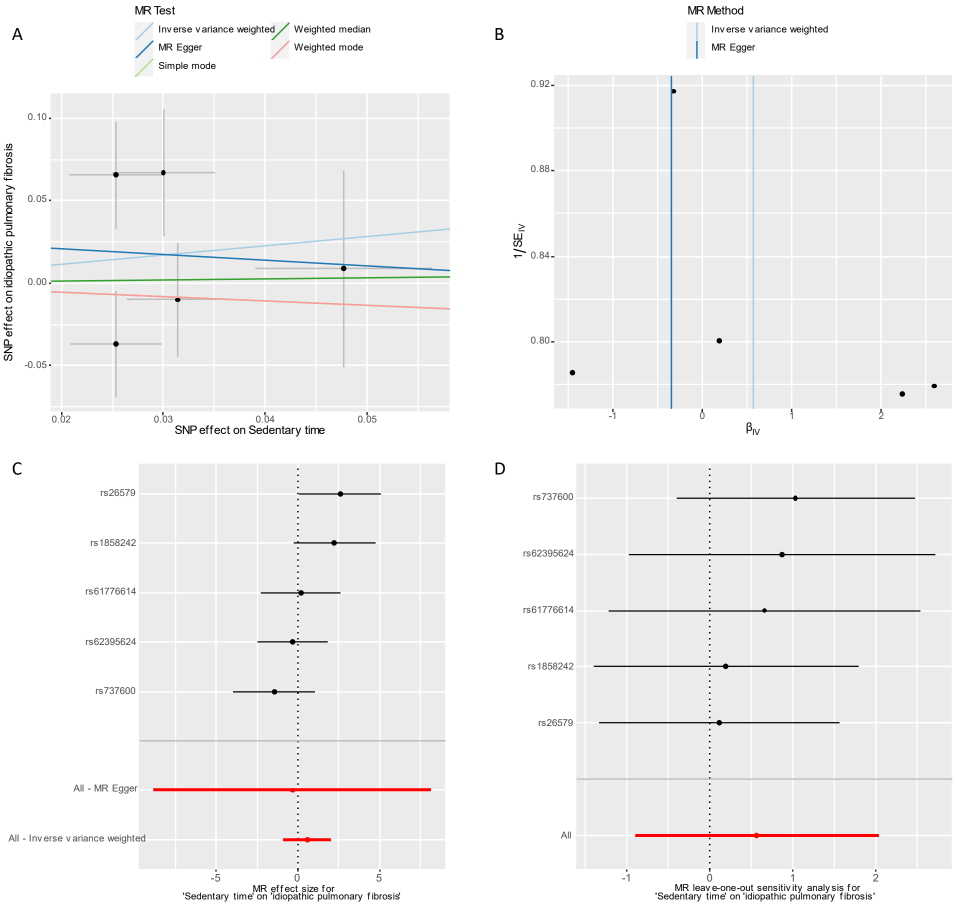

Figure S3. Scatter plot(A), Funnel plot(B), forest plot(C) and leave-one-out analysis(D) for sedentary time on idiopathic pulmonary fibrosis.
